# Polymerised type I collagen modifies the physiological network of post‐acute sequelae of COVID‐19 depending on sex: a randomised clinical trial

**DOI:** 10.1002/ctm2.1436

**Published:** 2023-10-29

**Authors:** Janette Furuzawa‐Carballeda, Paola Vanessa Olguín‐Rodríguez, Gonzalo Torres‐Villalobos, Antonio Barajas‐Martínez, Vania Jocelyn Martínez‐Garcés, Ruben Fossion, Marco Antonio Martínez‐Rivera, Elizabeth Ibarra‐Coronado, Geraldine Tello‐Santoyo, Octavio Abraham Bureos Lecona, Silvia Méndez‐Flores, Kenia Ilian Rivas‐Redonda, Eric Ochoa‐Hein, Elizabeth Olivares‐Martínez, Diego Francisco Hernández‐Ramírez, Ana Leonor Rivera

**Affiliations:** ^1^ Departamento de Inmunología y Reumatología Instituto Nacional de Ciencias Médicas y Nutrición Salvador Zubirán Mexico City Mexico; ^2^ Centro de Investigación en Ciencias Universidad Autónoma del Estado de Morelos Cuernavaca Morelos Mexico; ^3^ Centro de Ciencias de la Complejidad Universidad Nacional Autónoma de México Mexico City Mexico; ^4^ Departamento de Cirugía y Cirugía Experimental Instituto Nacional de Ciencias Médicas y Nutrición Salvador Zubirán Mexico City Mexico; ^5^ Plan de Estudios Combinados en Medicina (PECEM‐MD/PhD) Facultad de Medicina Universidad Nacional Autónoma de México Mexico City Mexico; ^6^ Instituto de Ciencias Nucleares Universidad Nacional Autónoma de México Mexico City Mexico; ^7^ Departamento de Fisiología, Facultad de Medicina Universidad Nacional Autónoma de México Mexico City Mexico; ^8^ Departamento de Biología Celular y Tisular Facultad de Medicina Universidad Nacional Autónoma de México Mexico Mexico; ^9^ Doctorado en Ciencias Biomédicas Universidad Nacional Autónoma de México Mexico City Mexico; ^10^ Departamento de Dermatología Instituto Nacional de Ciencias Médicas y Nutrición Salvador Zubirán Mexico City Mexico; ^11^ Departamento de Epidemiología Hospitalaria Instituto Nacional de Ciencias Médicas y Nutrición Salvador Zubirán Mexico Mexico

Dear Editor,

Current research focuses on understanding the physiological recovery process from COVID‐19 at a systemic level. Post‐acute sequelae COVID‐19 (PASC) comprise persistent and/or delayed effects beyond 4 weeks after symptom onset.^1^ These symptoms may exhibit a higher incidence in one gender compared to the other.^1^, ^2^ Previous studies indicate that young women have more interconnected, dense and clustered physiological networks than young men.^3^ Females generally exhibit a better prognosis than males for acute systemic diseases such as COVID‐19, attributed to their more robust physiological networks.^4^ However, little is known about the impact of PASC, low‐grade inflammation^5^ and sex differences on this systemic structure.

Polymerised type I collagen (PTIC) is a potential drug for treating COVID‐19 patients, proven to be effective against rheumatoid arthritis,^6^, ^7^ and is being investigated in multiple preclinical studies for other diseases.

We analysed the data from a double‐blind, randomised controlled trial of PTIC,^6^, ^7^, ^8^, ^9^ as an immune down‐regulator, in COVID‐19. We examined the dynamics of the physiological network, which characterises system‐wide modifications during post‐acute recovery.

Between August 31 and November 7, 2020, 89 unvaccinated adult outpatients with Polymerase Chain Reaction (PCR)‐confirmed COVID‐19 were treated at the Instituto Nacional de Ciencias Médicas y Nutrición Salvador Zubirán. They were randomly allocated to receive either 1.5 mL of PTIC intramuscularly every 12 h for 3 days, followed by every 24 h for 4 days (*n* = 45), or a matching placebo (*n* = 44) and followed for 12 weeks. Additional experimental details can be found in Méndez‐Flores et al. (2022).^8^ The anonymised database can be accessed at https://www.c3.unam.mx/pasc/.

Our analysis focused only on patients who had completed all assessments in both the PTIC group (*n* = 20, females = 8, males = 12) and the placebo group (*n* = 17, females = 10, males = 7). Vital signs, anthropometric measurements and blood samples were collected on days 1 (baseline), 8 (first‐day post‐treatment), 15 (day 8 post‐treatment) and 97 (day 90 post‐treatment). The cytokines and chemokines were classified by function (Table S1). Sample was stratified into four groups: men or women who received either a placebo or PTIC treatment. Detailed demographic, clinical and chemical information can be found in Tables S2 and S3. The immune response timeline of the control group is depicted in Figure S1.

To assess the treatment effects, we obtained relative values for each variable and all groups using Equation (1) provided in Supporting information. Figure S2 illustrates the relative values on day 1 post‐treatment, which were close to zero in both groups. Most variables decreased on days 8 and 90 post‐treatment, indicating recovery. We observed significant differences between groups (Figure S3C) on first‐day post‐treatment in IP‐10, IL‐8, CRP, basophil to lymphocyte ratio (*p* < .01), eosinophil to lymphocyte ratio, and IL‐1Ra (*p* < .05). On day 8 post‐treatment, there were significant differences in eosinophil count and eosinophil to lymphocyte ratio (*p* < .05). On day 90 post‐treatment, there were significant differences in IL‐2Ra (*p* < .01), IL‐13, eosinophils, and eosinophils to lymphocyte ratio (*p* < .05). Our findings suggest that PTIC reduced the hyperinflammation associated with COVID‐19.

In the groups of male participants, significant changes were observed in the essential mediators for inflammation regulation (Figure S4A,B). However, in the groups of female participants, only a few variables showed modifications compared to placebo (Figure S4C,D). Specifically, in the PTIC group from 8 to 90 days post‐treatment, only one variable was detected: ferritin in the male group and MIP‐1a in the female group. In contrast, a greater number of variables remained altered in the placebo group. These findings suggest that the downregulation of hyperinflammation by PTIC commenced early and persisted over time.

Figure 1 illustrates sex‐dependent changes in relative values. On the first‐day post‐treatment, the female PTIC group exhibited a decrease in the cytokines IL‐1Ra, IL‐8 and IP‐10, associated with inflammation in severe COVID‐19.^10^ In contrast, the placebo group showed increases in the inflammatory biomarkers TNF‐α, PDGF‐BB, SDF‐1α and M‐CSF, and eosinophils. Among men in the PTIC group, SCGF‐β and IP‐10 increased, while IL‐Ra and IL‐8 decreased. In the placebo group, only IFN‐γ decreased. Additional variables were found to differ at 8‐ and 90‐days post‐treatment, as expected due to the anticipated recovery.

**FIGURE 1:**
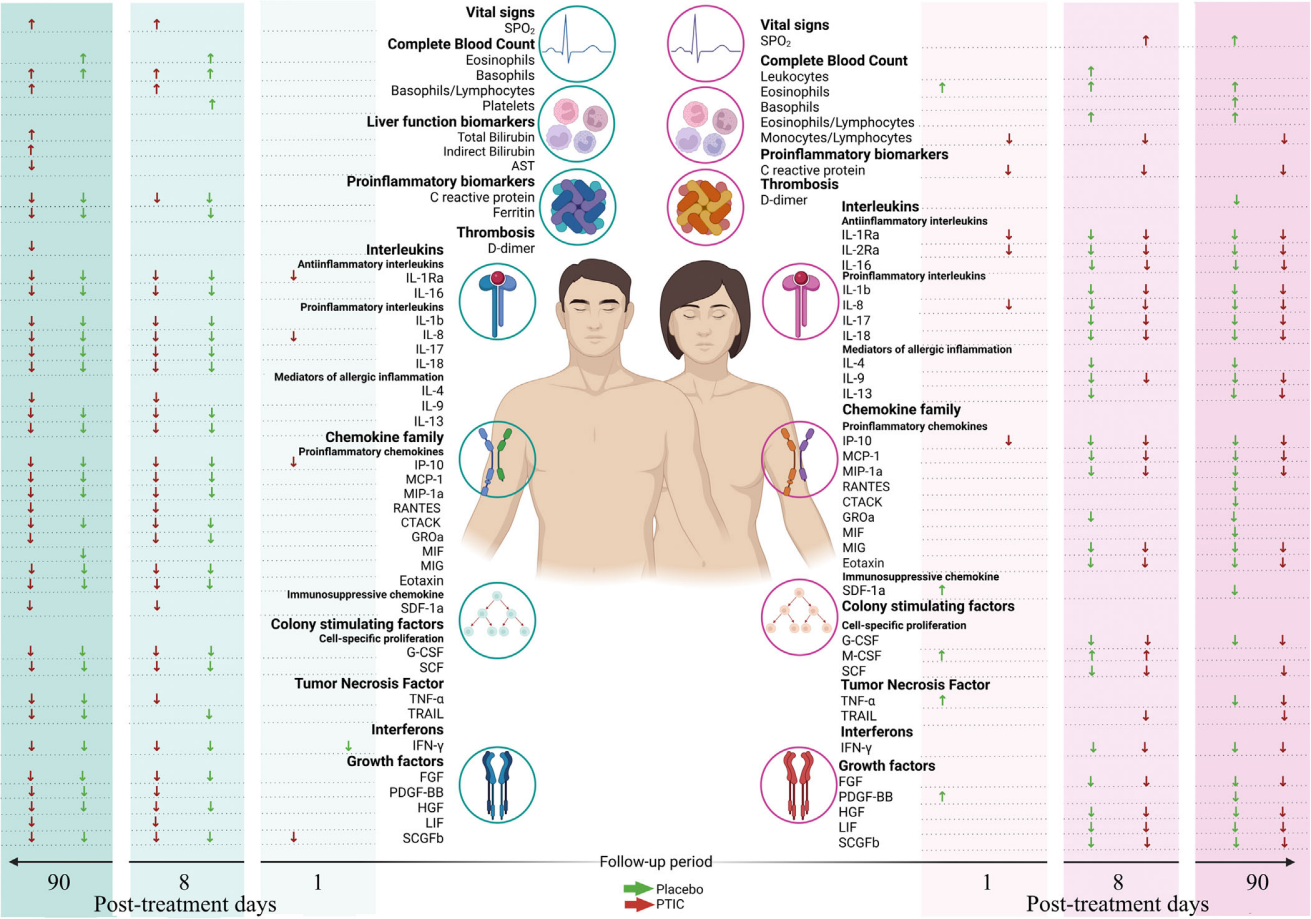
Significant relative physiological variables on days 1, 8 and 90 post‐treatment, distinguishing between men (left‐hand side) and women (right‐hand side) and also distinguishing between the placebo group (green‐coloured arrows) and the PTIC treatment group (red‐coloured arrows). Shown are changes in magnitude for each variable compared with the baseline (increases represented by upwards arrows and decreases by downwards arrows). PTIC, Polymerised type I collagen.

In contrast to women, PTIC administration in men only affected transiently a few variables (Figure S5 and Table S3).

Network adjacency was calculated using a Spearman correlation matrix, filtered with *p* < .05. Figure 2A–D shows the physiological networks for the male and female groups, as well as for the full dataset (see Figure S6). On the first‐day post‐treatment, exclusively in PTIC groups, a high network transitivity and density between immunological variables were observed (Table S4).

**FIGURE 2:**
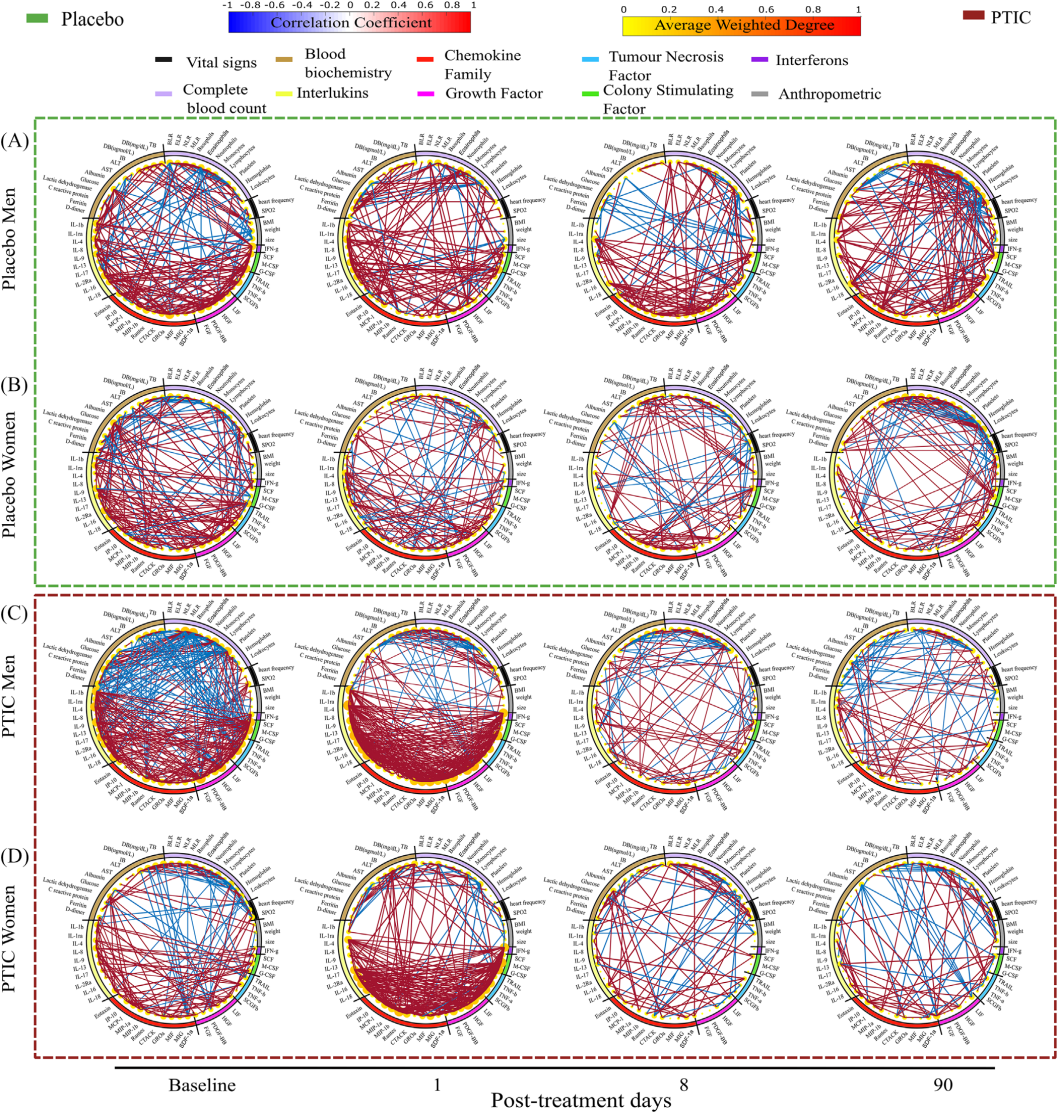
Physiological networks for (A) the male placebo group, (B) the female placebo group, (C) the male PTIC treatment group and (D) the female PTIC treatment group, at baseline and for days 1, 8 and 90 post‐treatment. PTIC, Polymerised type I collagen.

Figure 3 illustrates the declining correlation between baseline and post‐treatment days, which was faster in the PTIC groups. Treatment is crucial for the recovery process, particularly in the case of the female group. Women in the PTIC group exhibited a stronger response, evidenced by a lower correlation of the network between baseline and the first‐day post‐treatment (rho = 0.17) as compared to the male group (rho = 0.36).

**FIGURE 3:**
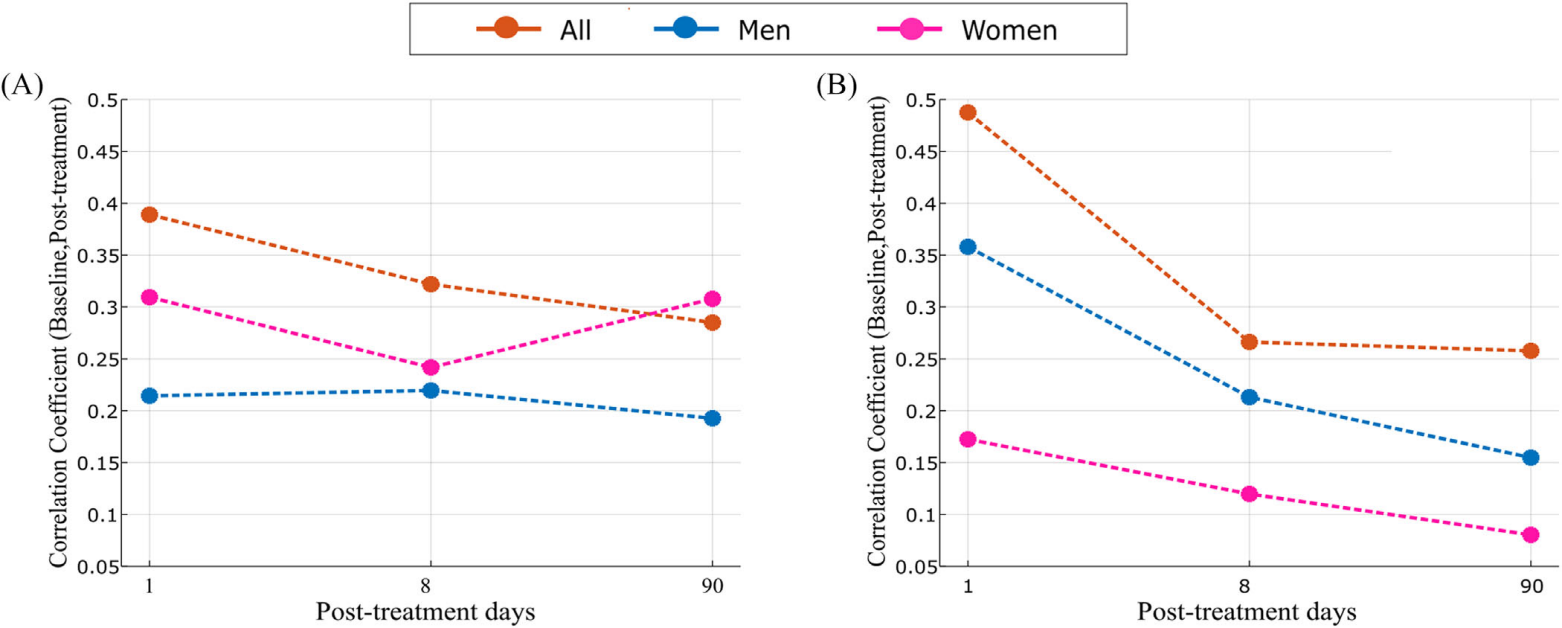
Correlation coefficients between physiological networks at baseline and days 1, 8 and 90 post‐treatment for (A) the placebo group and (B) the PTIC group. PTIC, Polymerised type I collagen.

This study confirms previous findings that women with COVID‐19 experience better outcomes when they receive treatment. In the placebo group, women exhibited a higher correlation between their physiological networks at baseline and 90 days post‐treatment, indicating a slower recovery.

In summary, physiological networks can serve as a valuable tool for evaluating the recovery of PASC. The topological features of the physiological networks of men are more vulnerable to acute disturbances than those of women. However, their flexibility increases the expediency of the return to normalcy from an altered state. This would be advantageous when considering long‐term recovery from a post‐acute infection. Administration of PTIC reduced hyperinflammation, leading to a decrease in the correlation between physiological networks at baseline and post‐treatment days in the PTIC group. In contrast, the placebo group of women continued to exhibit a strong similarity to the network at baseline, even after a 90‐days post‐treatment. Immunomodulatory drugs, such as PTIC, may necessitate an extended course in women with a robust physiological network.

## Supporting information

Supporting Information

Supporting Information

Supporting Information

Supporting Information

Supporting Information

Supporting Information

Supporting Information
