# Supplementary material for: Polymerised type I collagen modifies the physiological network of post‐acute sequelae of COVID‐19 depending on sex: a randomised clinical trial
Source: Clin Transl Med. 2023 Oct 29;13(11):e1436. doi: 10.1002/ctm2.1436 (PMC10613754; doi:10.1002/ctm2.1436)
Supplement: Supplementary file 1 — Supporting Information [file CTM2-13-e1436-s007.docx]

**Supporting Information**

Janette Furuzawa-Carballeda^1+^, Paola V. Olguín-Rodríguez^2,3+^, Gonzalo Torres-Villalobos^4+^, Antonio Barajas-Martínez^3+^, Vania J. Martínez-Garcés^5^, Ruben Fossion^3,6^, Marco A. Martínez-Rivera^5^, Elizabeth Ibarra-Coronado^7^, Geraldine Tello-Santoyo^8^, Octavio Lecona^9^, Silvia Méndez-Flores^10^, Kenia Ilian Rivas-Redonda^1^, Eric Ochoa-Hein^11^, Elizabeth Olivares-Martínez^1^, Diego F. Hernández-Ramírez^1^, Ana Leonor Rivera^3,6,*^

^1^ Departamento de Inmunología y Reumatología, Instituto Nacional de Ciencias Médicas y Nutrición Salvador Zubirán, Mexico City, Mexico.

^2^ Centro de Investigación en Ciencias, Universidad Autónoma del Estado de Morelos, Cuernavaca, Morelos, Mexico.

^3^ Centro de Ciencias de la Complejidad, Universidad Nacional Autónoma de México, Mexico City, Mexico.

^4^ Departamento de Cirugía y Cirugía Experimental, Instituto Nacional de Ciencias Médicas y Nutrición Salvador Zubirán, Mexico City, Mexico.

^5^ Plan de Estudios Combinados en Medicina (PECEM-MD/PhD), Facultad de Medicina, Universidad Nacional Autónoma de México, Mexico City, Mexico.

^6^ Instituto de Ciencias Nucleares, Universidad Nacional Autónoma de México, Mexico City, Mexico.

^7^ Departamento de Fisiología, Facultad de Medicina, Universidad Nacional Autónoma de México, Mexico City, Mexico.

^8^ Departamento de Biología Celular y Tisular, Facultad de Medicina, Universidad Nacional Autónoma de México, Mexico City, Mexico.

^9^ Doctorado en Ciencias Biomédicas, Universidad Nacional Autónoma de México, Mexico City, Mexico.

^10^ Departamento de Dermatología, Instituto Nacional de Ciencias Médicas y Nutrición Salvador Zubirán, Mexico City, Mexico.

^11^ Departamento de Epidemiología Hospitalaria, Instituto Nacional de Ciencias Médicas y Nutrición Salvador Zubirán, Mexico City, Mexico.

**Correspondence Author**

*Ana Leonor Rivera,

Corresponding address: Circuito Exterior s/n, Ciudad Universitaria, Col. Universidad Nacional Autónoma de México, Alcaldía Coyoacán, Apartado Postal 70-543, C.P. 04510, CDMX, México.

Email: ana.rivera@nucleares.unam.mx

# METHODS



**Experimental design**



The dataset was provided by the “Instituto Nacional de Ciencias Médicas y Nutrición Salvador Zubirán”, a Level-3 hospital in Mexico City. The institution obtained approval for the study protocol (INCMNSZ, reference no. IRE 3412-20-21-1) in accordance with the WMA Declaration of Helsinki. This double-blind, randomized, placebo-controlled clinical trial was registered with the ClinicalTrials.gov identifier NCT04517162. Detailed experimental information can be found in Méndez-Flores et al.^1^ A total of eighty-nine adult outpatients with confirmed COVID-19 diagnosis (mild to moderate disease not requiring hospitalization) were recruited from August 31 to November 7, 2020, and followed for 12 weeks. None of the patients had been vaccinated against SARS-CoV-2. Informed consent was obtained from all patients. They were randomly assigned to receive either 1.5 ml of PTIC intramuscularly every 12 hours for 3 days and then every 24 hours for 4 days (n = 45) or a matching placebo (n = 44). Concomitant therapy was limited to acetaminophen or acetylsalicylic acid. The primary outcome measure was a reduction in the level of interferon-gamma inducible protein-10 (IP-10) by at least 50% relative to the baseline. Secondary outcomes included oxygen saturation of 92% or above while breathing ambient air and the duration of symptoms. Symptom severity was graded as follows: 0 = absent, 1 = mild, 2 = moderate, and 3 = severe. Two comparisons were made for symptom duration and intensity: one at 1, 8, and 90 post-treatment days compared to baseline in each group, and the other between the PTIC vs. placebo groups. Participants self-reported their responses for the secondary outcomes at 1, 8-, and 90 post-treatment days after randomization. On these post-treatments, 1 8, and 90, the journal and health status of each participant were double-checked through telephone and in-person review by a physician. Participants and study personnel actively monitored and reported adverse and major adverse events for the 90 days post-treatment following the first dosage of either PTIC or placebo.

# Physiological Variable Measurement



The anonymized database containing the physiological variables measured for all participants in this study can be obtained from J.F.-C. ([jfuruzawa@gmail.com](mailto:jfuruzawa@gmail.com)) and is available at <https://www.c3.unam.mx/pasc/>. In this study, we included only non-hospitalized patients who underwent all assessments of serum cytokines and chemokines in each group: the PTIC group (n=20, female= 8, male=12) and the placebo group (n=17, female= 10, male=7). Vital signs (heart rate and oxygen saturation), anthropometric measures (weight and body mass index, (BMI)), and blood samples were taken from study participants on days 1 (baseline), 8 (day 1 post-treatment), 15 (day 8 post-treatment) and 97 (day 90 post-treatment). Laboratory data were collected from each patient in the study (Table 1). Cytokine and chemokine concentrations were measured using the Hu Cyto screening panel 48-Plex kit (Bio-Rad®, Berkeley, Calif.); further experimental details can be found in the previous work[^1^](https://docs.google.com/document/d/1povTN0oXCNyOno9_sfQugmZ2drqwnS7s/edit#heading=h.49x2ik5). The cytokines and chemokines were categorized based on their function (Table 1) and the immune response timeline (Figure S1).

# Statistical Analysis



To assess treatment effects, we compared the relative values for each physiological variable to the baseline using

the formula:

$-1\leq\frac{V_{post-treatment}-V_{basal}}{V_{post-treatment}+V_{basal}}\leq1$ (1)

Values close to zero indicate no differences in variables between the baseline and post-treatment days (which means there is no apparent health improvement in that value). Values of -1 indicate that the magnitude of variables during post-treatment was less than at the baseline (*V_post_*_−_*_treatment_* ≤ *V_basal_*), while values close to 1 indicate that the magnitude during post-treatment days was larger than at the baseline (*V_post_*_−_*_treatment_* ≥ *V_basal_*). We employed a two-way ANOVA with Bonferroni correction by groups to detect the relative change of each physiological variable, stratified by sex, from baseline to post-treatment between the PTIC and placebo groups. To determine the changes in physiological variables between the placebo and PTIC groups during the assessment days, a volcano plot was generated, plotting the p-value of the statistical significance test against the magnitude of change (mean rank difference).

# Physiological network analysis



Physiological networks are a new paradigm for analyzing and conceptualizing interactions between multiple physiological systems. The use of physiological networks in healthy subjects to characterize and detect physiological states such as sleep phases changes in the aging processes and pathological conditions ^2,3,4,5^. This idea was extended to punctual data by Barajas et al., ^6,7^

The physiological network for each group on all study days was constructed following the methodology described by Barajas et al.^6,7^ To determine interactions between physiological variables, Spearman correlation matrices were calculated and filtered to include only correlation coefficients with a p-value $\leq$0.05, indicating statistically significance. The size and color of the nodes in the network represent the average weighted degree, which is calculated by averaging the correlation coefficients of each node with all the nodes. In essence, the correlation strength of the nodes reflects their importance in the network. The color and width of the edges indicate the sign and strength of the significant correlations between each variable pair. Variables were grouped by function and arranged clockwise, following their order in the database. To quantify changes in the physiological network due to the treatment, the correlation coefficient and the Mann-Whitney-Wilcoxon rank test were evaluated between baseline and post-treatment days.

**References:**

1. Méndez-Flores S, Priego-Ranero Á, Azamar-Llamas D, et al. Effect of polymerized type I collagen on hyperinflammation of adult outpatients with symptomatic COVID-19. *Clin Transl Med* 2022; 12(3).

1. Bashan, A., Bartsch, R. P., Kantelhardt, J. W., Havlin, S., & Ivanov, P. C. “Network physiology reveals relations between network topology and physiological function,” *Nature communications*, *3*(1), 702 (2012).
2. Ivanov, P. C. (2021). The new field of network physiology: building the human physiolome. *Frontiers in Network Physiology*, *1*, 711778.
3. Ivanov, P. C., Wang, J. W., Zhang, X., & Chen, B. (2021). The new frontier of network physiology: emerging physiologic states in health and disease from integrated organ network interactions. *2019-20 MATRIX Annals*, 237-254.
4. Nakazato, Y., Shimoyama, M., Cohen, A. A., Watanabe, A., Kobayashi, H., Shimoyama, H., & Shimoyama, H. (2023). Intercorrelated variability in blood and hemodynamic biomarkers reveals physiological network in hemodialysis patients. *Scientific Reports*, *13*(1),
5. Barajas-Martínez, A., Easton, J. F., Rivera, A. L., Martínez-Tapia, R., De la Cruz, L., Robles-Cabrera, A., & Stephens, C. R. (2020). Metabolic physiological networks: the impact of age. *Frontiers in Physiology*, *11*, 587994.
6. Barajas-Martínez, A., Ibarra-Coronado, E., Fossion, R., Toledo-Roy, J. C., Martínez-Garcés, V., López-Rivera, J. A., ... & Rivera, A. L. (2021). Sex differences in the physiological network of healthy young subjects. *Frontiers in Physiology*, *12*, 678507.

The cytokines and chemokines were categorized based on their function

| **Categories** | **variables** | |
| --- | --- | --- |
| Complete blood count | Leukocytes  Hemoglobin  Platelets  Lymphocytes  Monocytes Neutrophils  Eosinophils  Basophils | Monocytes/Lymphocytes  Neutrophils/Lymphocytes  Eosinophils/Lymphocytes  Basophils/Lymphocytes |
| Blood biochemistry | Total bilirubin  Direct bilirubin  Indirect bilirubin  Albumin (ALT)  Astrocytes (AST) | |
|  | Glucose  Lactic dehydrogenase  C reactive protein  Ferritin  D-dimer | |
| Interleukins | Hu IL-1β  Hu IL-1Ra  Hu IL-4  Hu IL-8  Hu IL-9  Hu IL-13  Hu IL-17  Hu IL-2Ra  Hu IL-16  Hu IL-18 | |
| Chemokine family | Hu Eotaxin  Hu IP-10  Hu MCP-1  Hu MIP-1α  Hu MIP-1β  Hu Rantes  Hu CTACK  Hu GROα  Hu MIF  Hu MIG  Hu SDF-1α | |
| growth factors | Hu FGF  Hu PDGF-BB  Hu VEGF  Hu HGF  Hu LIF  Hu SCGFβ | |
| colony stimulating | Hu M-CSF  Hu SCF | |
| tumor necrosis factor | Hu TNF-α  Hu TNF-β  Hu TRAIL | |
| Interferons | Hu IFN-γ | |

Table S1: Laboratory data and serum cytokine and chemokines.

Detailed demographic, clinical, and chemical information

|  | **Baseline** | | | **1-day post-treatment** | | | **8-day post-treatment** | | | **90-day post-treatment** | | |
| --- | --- | --- | --- | --- | --- | --- | --- | --- | --- | --- | --- | --- |
|  | **Women**  **(N= 8)** | **Men**  **(N= 12)** | ***P*** | **Women**  **(N= 8)** | **Men**  **(N= 12)** | ***P*** | **Women**  **(N= 8)** | **Men**  **(N= 12)** | ***P*** | **Women**  **(N= 8)** | **Men**  **(N= 12)** | ***P*** |
| **Demographics** | | | | | | | | | | | | |
| Age (years), mean±SD  Median  Range | 48.1±18.8  40.0  29 –79 | 48.7±13.0  47.0  19–64 | 0.940 |  |  |  |  |  |  |  |  |  |
| BMI (kg/m2), mean±SD  Median  Range | 29.1±4.2  28.2  24.8–38.3 | 28.4±3.1  28.5  23.1–33.2 | 0.702 |  |  |  |  |  |  |  |  |  |
| pSO2<92% (%) | 1 (13) | 4 (33) | 0.6027 | 0 (0) | 2(17) | 0.4947 | 0 (0) | 0 (0) | 1.00 | 0 (0) | 0 (0) | 1.00 |
| pSO2; mean±SD  Median  Range | 92.0±4.2  94.0  84-96 | 92.5±1.6  92.0  91-96 | 0.4333 | 94.3±2.0  94.0  92-97 | 93.3±1.7  93.0  91-96 | 0.290 | 95.3±1.6  95.0  92-97 | 93.8±1.6  94.0  92-97 | 0.071 | 95.1±2.4  95.0  92-98 | 95.2±2.6  94.5  92-100 | 0.972 |
| **Laboratory variables** | | | | | | | | | | | | |
| Complete blood count |  |  |  |  |  |  |  |  |  |  |  |  |
| Leukocyte count (x10^3/µL), mean±SD  Median  Range | 6.7±1.5  6.9  4.0–8.0 | 5.8±2.5  5.4  2.8-12.4 | 0.153 | 6.8±1.4  6.5  5.2-9.3 | 5.9±1.4  6.1  3.6-8.3 | 0.169 | 6.7±1.0  6.8  5.4-7.9 | 6.5±1.3  6.5  4.8-9.6 | 0.775 | 7.3±0.9  7.2  5.9-8.5 | 6.4±1.5  6.5  3.7-8.3 | 0.152 |
| Hemoglobin (g/dL), mean±SD  Median  Range | 13.9±1.5  14.1  11.9-15.5 | 16.8±1.9  16.9  14.0-20.0 | **0.0021** | 13.9±1.2  14.1  11.9-15.5 | 16.4±1.3  16.2  14.7-19.3 | **0.0005** | 13.8±1.4  13.9  11.2-15.6 | 16.2±0.7  16.1  15.2-17.6 | **0.00008** | 14.4±1.4  14.5  12.0-15.9 | 16.6±1.2  16.4  14.2-19.4 | **0.00130** |
| Platelets (K/µL), mean±SD  Median  Range | 289.3±55.4  298.5  218.0-376.0 | 283.8.±207.5  216.5  150.0-910.0 | 0.165 | 332.0±118.8  339.0  151.0-419.0 | 329.4±128.8  290.0  223.0-642.0 | 0.964 | 319.4±95.0  343.0  172.0-469.0 | 281.8±85.9  274.5  166.0-439.0 | 0.408 | 302.5±98.0  315.0  169.0-460.0 | 254.2±50.4  262.5  179.0.-352.00 | 0.163 |
| Lymphocyte count (%), mean±SD  Median  Range | 28.5±10.1  31.1  13.2–42.8 | 27.4±11.6  27.7  8.1-43.5 | 0.827 | 33.0±6.0  33.9  19.5-40.5 | 30.7±7.6  32.3  17.4-42.2 | 0.481 | 32.9±5.6  35.0  20.4-35.8 | 30.7±9.3  30.2  15.1-46.4 | 0.547 | 34.1±3.0  34.5  29.2-39.0 | 30.6±8.0  30.0  18.1-41.5 | 0.253 |
| Neutrophil count (%), mean±SD  Median  Range | 61.6±12.1  57.5  46.3–78.2 | 63.1±11.1  62.3  48.1–80.4 | 0.778 | 57.7±6.2  57.0  49.8-71.0 | 58.9±7.1  57.8  49.0-69.7 | 0.706 | 57.7±5.2  56.5  51.7-69.0 | 59.0±8.8  58.6  44.9-71.2 | 0.718 | 56.2±3.7  55.7  51.6-64.6 | 60.0±7.3  61.1  50.5-71.3 | 0.202 |
| Neutrophil-lymphocyte ratio (NLR), mean±SD  Median  Range | 2.7±1.7  1.8  1.1-5.8 | 3.3±2.8  2.3  1.1-9.9 | 0.643 | 1.9±0.7  1.7  1.2-3.6 | 2.1±0.8  1.8  1.2-4.0 | 0.787 | 1.8±0.6  1.6  1.7-3.4 | 2.2±1.1  1.9  1.0-4.7 | 0.512 | 1.7±0.3  1.6  1.3-2.2 | 2.2±0.9  2.0  1.2-3.7 | 0.134 |
| **Liver function test (LFT)** | | | | | | | | | | | | |
| Total bilirubin (mg/dL), mean±SD  Median  Range | 0.5±0.1  0.5  0.3-0.7 | 0.7±0.3  0.6  0.4–1.3 | 0.107 | 0.6±0.2  0.6  0.4-0.8 | 0.9±0.3  0.8  0.4-1.1 | **0.0322** | 0.7±0.4  0.6  0.3-1.3 | 0.8±0.3  0.8  0.4-1.1 | 0.501 | 0.7±0.3  0.6  0.3-1.0 | 1.0±0.4  0.8  0.5-1.8 | 0.123 |
| Direct bilirubin (mg/dL), mean±SD  Median  Range | 0.1±0.0  0.1  0.0-0.2 | 0.2±0.10  0.1  0.1-0.2 | 0.461 | 0.1±0.0  0.1  0.1-0.2 | 0.1±0.1  0.1  0.1-0.2 | 0.137 | 0.1±0.0  0.1  0.1-0.2 | 0.1±0.0  0.1  0.1-0.2 | 0.907 | 0.1±0.0  0.1  0.1-0.2 | 0.1±0.0  0.1  0.1-0.2 | 0.312 |
| Indirect bilirubin (mg/dL), mean±SD  Median  Range | 0.4±0.1  0.4  0.2–0.6 | 0.6±0.2  0.5  0.3–1.0 | 0.0877 | 0.5±0.1  0.5  0.3-0.7 | 0.7±0.2  0.7  0.3-1.0 | **0.0259** | 0.6±0.3  0.5  0.3-1.1 | 0.7±0.2  0.7  0.3-1.0 | 0.444 | 0.5±0.5  0.5  0.2-0.9 | 0.8±0.4  0.7  0.3-1.6 | 0.105 |
| Aminotransferase, serum aspartate (AST) (U/L), mean±SD  Median  Range | 27.5±19.5  23.9  11.0-83.0 | 30.9±12.3  29.0  15.0-59.8 | 0.104 | 19.7±4.9  17.0  12.0-27.0 | 27.2±9.2  24.7  17.0-51.0 | **0.011** | 16.0±2.9  18.00  12.0-22.0 | 25.3±15.9  20.5  12.0-70.0 | **0.037** | 17.5±8.6  17.5  2.8-33.0 | 21.8±6.9  20.0  13.0-34.0 | 0.234 |
| Aminotransferase, serum alanine (ALT) (U/L), mean±SD  Median  Range | 26.4±14.0  27.6  9.8–92.0 | 42.9±33.1  34.0  9.0–129.8 | 0.132 | 22.94±17.6  16.0  9.0-60.0 | 41.3±19.8  42.5  14.0-88.0 | **0.0475** | 14.1±5.3  15.0  6.0-21.0 | 31.7±13.4  28.5  10.0-60.0 | **0.0026** | 18.1±10.4  15.0  5.0-39.0 | 24.8±11.7  22.0  12.0-50.0 | 0.113 |
| Albumin (g/dL), mean±SD  Median  Range | 4.3 ± 0.5  4.3  3.5 - 5.1 | 4.5 ± 0.3  4.5  4.0-5.1 | 0.348 | 4.4±0.3  4.6  3.9-4.8 | 4.1±0.8  4.2  1.9-5.1 | 0.280 | 4.5±0.5  4.4  4.0-5.6 | 4.5±0.4  4.5  3.8-5.1 | 0.721 | 4.7±0.3  4.7  4.2-5.1 | 4.6±0.4  4.7  4.0-5.2 | 0.871 |
| Fasting glucose (mg/dL)  Mean±SD  Median  Range | 91.8±12.6  86.5  79-110 | 133.0±90.2  108.0  70-386 | 0.153 | 98.5±12.8  98.5  81-120 | 121.6±76.5  96.5  84-361 | 0.728 | 97.5±10.6  93.0  88-116 | 117.7±58.8  100.0  85-297 | 0.699 | 92.4±6.0  93.0  84-99 | 111.5±55.8  96.0  80-286 | 0.354 |
| Lactate dehydrogenase (LDH) (U/L)  Mean±SD  Median  Range | 168.3±69.0  162.0  97-269 | 175.3±64.0  156.5  116-303 | 0.758 | 136.4±35.0  138.0  95-179 | 157.1±61.4  140.5  113.0-338.0 | 0.537 | 128.5±28.3  124.0  91.0-169.0 | 137.3±22.3  135.5  94.0-169.0 | 0.449 | 144.1±24.7  156.00  104.0-169.0 | 149.5±24.5  151.0  109.0-192.0 | 0.638 |
| C-reactive protein (mg/dL)  mean±SD  Median  Range | 3.0±5.5  1.3  0.1-16.5 | 1.9±2.1  1.3  0.1-7.2 | 0.934 | 0.3±0.1  0.3  0.1-0.4 | 0.5±0.4  0.4  0.1-1.3 | 0.220 | 0.2±0.1  0.2  0.0-0.4 | 0.4±1.0  0.1  0.1-3.6 | 0.132 | 0.3±0.1  0.4  0.1-0.4 | 0.2±0.3  0.1  0.0-1.1 | **0.020** |
| Ferritin (ng/mL)  mean±SD  Median  Range | 151.3±135.9  126.0  5.8-363.0 | 374.6±410.7  314.9  91.2-1614.4 | **0.045** | 95.5±76.9  96.3  3.4-201.6 | 303.1±300.9  218.7  91.1-1194.1 | **0.017** | 86.9±81.0  63.3  2.5-225.1 | 205.7±157.7  169.8  49.3-626.8 | **0.025** | 63.0±64.5  40.7  12.9-194.4 | 114.5±94.1  79.3  26.3-378.5 | **0.045** |
| D-dimer (ng/dL)  mean±SD  Median  Range | 3176.1±5267.5  570.5  192-15000 | 581.0±523.8  434.5  214-2074 | 0.123 | 1627.6±2556.4  517.5  191-7525 | 528.7±455.3  359.5  154-1885 | 0.700 | 1433.1±2094.5  470.5  191-6333 | 461.3±383.0  286.5  190-1387 | 0.217 | 571.0±560.4  438.5  169-1894 | 242.2±74.3  220.5  169-374 | 0.062 |
| **Number of comorbidities** | | | | | | | | | | | | |
| None, n, (%) | 0 (0) | 2 (17) | 0.4947 |  |  |  |  |  |  |  |  |  |
| One, n, (%) | 4 (50) | 0 (0) | **0.0144** |  |  |  |  |  |  |  |  |  |
| 2 or More, n, (%) | 4 (50) | 8 (83) | 0.6479 |  |  |  |  |  |  |  |  |  |
| **Clinical Comorbidities** | | | | | | | | | | | | |
| Overweight, n, (%) | 4 (50) | 7 (58) | 1.000 |  |  |  |  |  |  |  |  |  |
| Obesity, n, (%) | 3 (38) | 3 (25) | 0.6424 |  |  |  |  |  |  |  |  |  |
| Diabetes, n, (%) | 0 (0) | 2 (17) | 0.4974 |  |  |  |  |  |  |  |  |  |
| Dyslipidemia, n, (%) | 2 (25) | 3 (25) | 1.000 |  |  |  |  |  |  |  |  |  |
| Hypertriglyceridemia, n, (%) | 4 (50) | 8 (67) | 0.6479 |  |  |  |  |  |  |  |  |  |
| **Symptoms** | | | | | | | | | | | | |
| Dyspnea, n (%) | 3 (38) | 3 (25) | 0.6424 | 3 (15) | 1 (8) | 0.2745 | 0 (0) | 0 (0) | 1.000 | 1 (5) | 3 (25) | **0.044** |
| Cough, n (%) | 6 (75) | 10 (83) | 1.000 | 8 (100) | 5 (42) | **0.0147** | 6 (30) | 3 (25) | 0.0648 | 3 (15) | 3 (25) | 0.6424 |
| Chronic fatigue syndrome*  mean±SD  Median  Range | 4.9±4.1  5.5  0-10 | 3.8±2.9  4.0  0.0-8 | 0.509 | 2.8±3.3  2.0  0-9 | 2.6±3.0  1.0  0.0-7.0 | 0.967 | 2.4±1.8  3.0  0-5 | 2.1±2.3  1.0  0.0-6 | 0.753 | 1.5±1.2  1.5  0-3 | 1.6±2.0  1.0  0-6 | 0.690 |
| **Treatment** | | | | | | | | | | | | |
| Corticoids | 0 (0) | 0 (0) | 1.000 | 0 (0) | 0 (0) | 1.000 | 0 (0) | 0 (0) | 1.000 | 0 (0) | 0 (0) | 1.000 |
| Antibiotics | 6 (75) | 3 (25) | 0.0648 |  |  |  |  |  |  |  |  |  |
| Antipyretics | 6 (75) | 10 (83) | 1.000 |  |  |  |  |  |  |  |  |  |
| Antivirals | 1 (13) | 1 (8) | 1.000 |  |  |  |  |  |  |  |  |  |

BMI: body mass index; PTIC: polymerized type I collagen; pSO2: oxygen saturation; SD: standard deviation.

*Chalder fatigue scale: 4 or more qualifying for "caseness."

**TABLE S2** Demographic and clinical characteristics of the trial population under PTIC treatment

|  | **Baseline** | | | **1-day post-treatment** | | | **8-day post-treatment** | | | **90-day post-treatment** | | |
| --- | --- | --- | --- | --- | --- | --- | --- | --- | --- | --- | --- | --- |
|  | **Women**  **(N= 10)** | **Men**  **(N= 7)** | ***P*** | **Women**  **(N= 10)** | **Men**  **(N= 7)** | ***P*** | **Women**  **(N= 10)** | **Men**  **(N= 7)** | ***P*** | **Women**  **(N= 10)** | **Men**  **(N= 7)** | ***P*** |
| **Demographics** | | | | | | | | | | | | |
| Age (years), mean±SD  Median  Range | 43.3±9.5  43.5  31 –56 | 52.1±12.1  57.0  31–65 | 0.111 |  |  |  |  |  |  |  |  |  |
| BMI (kg/m2), mean±SD  Median  Range | 32.1±4.6  30.9  25.2–40.8 | 29.6±3.8  30.5  22.7–34.3 | 0.440 |  |  |  |  |  |  |  |  |  |
| pSO2<92% (%) | 3 (30) | 3 (43) | 0.6437 | 2 (20) | 3 (43) | 0.5928 | 2 (20) | 2 (29) | 1.000 | 1 (10) | 0 (0) | 1.000 |
| pSO2; mean±SD  Median  Range | 91.7±3.1  92.0  86-94 | 91.7±2.7  92.0  87-96 | 0.992 | 91.9±3.4  93.0  84-95 | 92.0±4.1  91.0  86-97 | 0.953 | 91.7±3.1  92.0  87-97 | 92.0±2.8  93.0  88-96 | 0.861 | 94.8±2.4  94.5  91-99 | 93.9±2.1  93.0  92-98 | 0.416 |
| **Laboratory variables** | | | | | | | | | | | | |
| Complete blood count | | | | | | | | | | | | |
| Leukocyte count (x10^3/µL), mean±SD  Median  Range | 5.6±2.1  4.8  3.7–9.7 | 6.2±3.5  4.9  3.0-12.5 | 0.625 | 6.6±1.4  6.7  4.9-9.1 | 6.5±2.3  5.7  4.9-11.4 | 0.525 | 7.1±1.2  7.2  5.1-8.9 | 7.0±1.4  6.6  5.8-9.7 | 0.960 | 6.6±1.5  6.7  4.6-9.5 | 6.9±1.8  6.2  5.0-9.3 | 0.751 |
| Hemoglobin (g/dL), mean±SD  Median  Range | 14.7±1.8  15.0  10.5-17.2 | 15.5±1.5  15.3  13.6-18.1 | 0.371 | 14.1±2.0  14.4  9.7-16.9 | 15.2±1.1  14.9  13.6-16.7 | 0.185 | 13.9±1.7  14.2  10.0-16.2 | 15.2±1.3  14.7  13.4-16.9 | 0.0943 | 14.2±1.54  14.2  11.2-16.7 | 15.6±0.9  15.5  14.3-17.1 | **0.480** |
| Platelets (K/µL), mean±SD  Median  Range | 264.0±137.4  239.0  118.0-568.0 | 243.3±100.3  251.0  134.0-436.0 | 0.739 | 339.6±148.7  288.5  153.0-605.0 | 324.0±78.9  321.0  284.0-440.0 | 0.804 | 382.8±203.1  310.0  169.0-875.0 | 338.3±56.4  312.0  284.0-440.0 | 0.770 | 305.9±128.4  299.5  150.0-579.0 | 252.3±37.8  237.0  218.0.-316.0 | 0.304 |
| Lymphocyte count (%), mean±SD  Median  Range | 33.2±11.4  31.1  21.1–54.0 | 25.4±9.8  23.3  15.1-40.3 | 0.165 | 30.5±7.2  31.2  17.0-42.0 | 23.8±9.2  25.0  6.8-35.7 | 0.113 | 31.5±6.0  31.2  23.4-39.6 | 26.7±8.2  28.7  12.6-35.9 | 0.180 | 36.0±7.8  35.8  26.4-49.3 | 30.3±7.2  31.2  17.8-38.2 | 0.151 |
| Neutrophil count (%), mean±SD  Median  Range | 57.9±10.6  58.7  39.0–70.2 | 65.5±9.5  70.5  51.8–73.6 | 0.148 | 59.5±8.0  59.8  48.9-72.5 | 66.6±9.6  65.6  50.7-81.7 | 0.120 | 58.2±6.7  58.2  48.7-66.2 | 62.8±8.4  63.1  50.5-76.3 | 0.228 | 54.4±8.0  56.0  41.9-64.4 | 59.2±6.6  57.4  51.3-71.9 | 0.207 |
| Neutrophil-lymphocyte ratio (NLR), mean±SD  Median  Range | 2.0±0.9  1.8  0.7-3.3 | 3.0±1.4  3.0  1.3-4.8 | 0.0915 | 2.1±0.9  1.9  1.2-4.3 | 3.9±3.6  2.6  1.4-12.0 | 0.143 | 1.9±0.6  1.9  1.2-2.8 | 2.8±1.6  2.2  1.4-6.1 | 0.205 | 1.6±0.5  1.6  0.9-2.4 | 2.1±0.9  1.8  1.4-4.0 | 0.168 |
| **Liver function test (LFT)** | | | | | | | | | | | | |
| Total bilirubin (mg/dL), mean±SD  Median  Range | 0.5±0.2  0.5  0.2-0.7 | 0.5±0.2  0.5  0.4–0.8 | 0.590 | 0.5±0.2  0.6  0.2-0.7 | 0.6±0.2  0.6  0.4-1.0 | 0.241 | 0.6±0.3  0.6  0.2-1.4 | 0.6±0.2  0.6  0.4-0.9 | 0.659 | 0.5±0.1  0.5  0.3-0.7 | 0.7±0.2  0.7  0.5-1.0 | **0.00768** |
| Direct bilirubin (mg/dL), mean±SD  Median  Range | 0.1±0.1  0.1  0.0-0.2 | 0.1±0.0  0.1  0.1-0.2 | 0.353 | 0.1±0.0  0.1  0.1-0.2 | 0.2±0.1  0.2  0.1-0.3 | 0.0781 | 0.1±0.1  0.1  0.0-0.2 | 0.1±0.0  0.1  0.1-0.2 | 0.879 | 0.1±0.0  0.1  0.1-0.2 | 0.1±0.0  0.2  0.1-0.2 | **0.0333** |
| Indirect bilirubin (mg/dL), mean±SD  Median  Range | 0.4±0.2  0.4  0.2–0.6 | 0.4±0.1  0.4  0.3–0.6 | 0.710 | 0.4±0.1  0.4  0.2-0.6 | 0.5±0.1  0.5  0.3-0.7 | 0.383 | 0.5±0.3  0.5  0.2-1.2 | 0.5±0.2  0.5  0.3-0.7 | 0.695 | 0.4±0.1  0.4  0.3-0.5 | 0.6±0.2  0.6  0.4-0.8 | **0.00725** |
| Aminotransferase, serum aspartate (AST) (U/L), mean±SD  Median  Range | 39.8±43.6  27.0  9.0–158.0 | 38.4±13.2  35.0  26.0-59.0 | 0.241 | 26.3±14.7  22.0  14.0-64.0 | 48.3±38.3  42.0  17.0-126.0 | 0.171 | 22.5±10.4  22.5  12.0-45.0 | 26.0±12.1  20.0  15.0-49.0 | 0.525 | 24.2±13.0  19.0  10.0-53.0 | 32.6±24.4  23.0  20.0-87.0 | 0.078 |
| Aminotransferase, serum alanine (ALT) (U/L), mean±SD  Median  Range | 44.0±33.0  35.5  12.7–120.0 | 39.9±27.5  31.0  12.0–97.0 | 0.696 | 33.8±16.5  27.0  15.0-60.0 | 56.4±57.7  29.0  20.0-178.0 | 0.434 | 28.4±10.1  29.0  15.0-46.0 | 32.4±13.8  28.0  18.0-52.0 | 0.496 | 32.2±17.0  29.5  13.0-73.0 | 26.6±13.0  22.0  15.0-54.0 | 0.351 |
| Albumin (g/dL), mean±SD  Median  Range | 4.2 ± 0.4  4.2  3.5 – 4.8 | 4.3 ± 0.2  4.2  4.1-4.6 | 0.576 | 4.0±0.4  3.9  3.6-4.8 | 4.1±0.4  4.0  3.4-4.6 | 0.782 | 4.1±0.3  4.1  3.7-4.8 | 4.2±0.3  4.2  3.6-4.5 | 0.818 | 4.2±0.2  4.2  3.9-4.6 | 4.4±0.2  4.5  4.0-4.7 | 0.0980 |
| Fasting glucose (mg/dL)  Mean±SD  Median  Range | 156.3±106  101.0  79-354 | 117.7±52.0  105.0  85-233 | 0.845 | 131.1±71.9  94.0  82-286 | 107.1±22.2  100.0  87-155 | 0.526 | 127.6±75.1  94.5  78-317 | 108.4±35.8  95.0  84.0-187.0 | 0.961 | 129.2±72.6  93.0  85-307 | 108.1±20.5  99.0  90.0-148.0 | 0.433 |
| Lactate dehydrogenase (LDH) (U/L)  Mean±SD  Median  Range | 166.4±24.5  160.5  125-204 | 179.7±45.5  169.0  130-271 | 0.445 | 164.4±29.1  158.5  127-222 | 201.0±90.6  182.0  121-397 | 0.380 | 163.6±29.1  159.0  114-252 | 187.6±105.8  162.0  104-422 | 0.992 | 159.5±22.2  159.5  125-189 | 166.9±21.6  165.0  141.0-197.0 | 0.507 |
| C-reactive protein (mg/dL)  mean±SD  Median  Range | 1.2±1.0  0.9  0.1-3.1 | 3.1±3.9  1.4  0.2-11.5 | 0.205 | 1.8±2.0  0.8  0.1-3.1 | 6.1±8.2  1.8  0.4-22.7 | 0.172 | 0.6±0.4  0.6  0.1-1.9 | 1.9±3.3  0.3  0.1-9.1 | 0.884 | 0.6±0.5  0.5  0.1-1.7 | 0.5±0.6  0.2  0.1-1.4 | 0.845 |
| Ferritin (ng/mL)  mean±SD  Median  Range | 215.1±386.0  63.2  5.6-1277.0 | 317.7±217.1  192.0  82.7-677.4 | 0.079 | 112.6±137.0  52.1  20.1-456.5 | 493.3±441.1  465.8  53.0-1420.2 | **0.006** | 77.2±71.0  50.2  13.3-201.5 | 309.7±268.6  246.7  29.8-860.1 | **0.015** | 43.5±46.4  24.5  3.9-137.8 | 106.4±85.9  66.3  22.8-276.5 | **0.040** |
| D-dimer (ng/dL)  mean±SD  Median  Range | 530.4±155.5  510.5  294-756 | 493.4±231.5  417.0  294-987 | 0.968 | 628±272.5  613.5  261-1225 | 1727.6±2621.4  917.0  295-7634 | 0.242 | 481.3±102.5  448.0  328-627 | 1702.4±2701.2  827.0  186-7764 | 0.283 | 389.6±274.3  315.5  186-1091 | 394.0±188.9  364.0  169-740 | 0.696 |
| Number of comorbidities | | | | | | | | | | | | |
| None, n, (%) | 0 (0) | 1 (14) | 0.4118 |  |  |  |  |  |  |  |  |  |
| One, n, (%) | 5 (50) | 1 (14) | 0.1516 |  |  |  |  |  |  |  |  |  |
| 2 or More, n, (%) | 5 (50) | 5 (71) | 0.6221 |  |  |  |  |  |  |  |  |  |
| **Clinical Comorbidities** | | | | | | | | | | | | |
| Overweight, n, (%) | 4 (40) | 2 (29) | 1.000 |  |  |  |  |  |  |  |  |  |
| Obesity, n, (%) | 6 (68) | 4 (57) | 1.000 |  |  |  |  |  |  |  |  |  |
| Diabetes, n, (%) | 4 (40) | 1 (14) | 0.3382 |  |  |  |  |  |  |  |  |  |
| Dyslipidemia, n, (%) | 0 (0) | 1 (14) | 0.4118 |  |  |  |  |  |  |  |  |  |
| Hypertriglyceridemia, n, (%) | 6 (60) | 3 (43) | 0.6372 |  |  |  |  |  |  |  |  |  |
| **Symptoms** | | | | | | | | | | | | |
| Dyspnea, n (%) | 5 (50) | 1 (14) | 0.3043 | 4 (40) | 2 (29) | 1.000 | 3 (30) | 1 (14) | 0.6029 | 4 (40) | 1 (14) | 0.3382 |
| Cough, n (%) | 9 (90) | 4 (57) | 0.2500 | 8 (80) | 5 (71) | 1.000 | 6 (60) | 4 (57) | 1.000 | 2 (20) | 1 (14) | 1.000 |
| Chronic fatigue syndrome*  mean±SD  Median  Range | 4.7±2.4  5.5  0-7 | 3.0±2.4  2.0  1-7 | 0.170 | 3.6±3.2  4.0  0-8 | 2.9±3.0  2.0  0.0-6.0 | 0.661 | 3.5±2.6  3.5  0-7 | 1.4±2.5  0.0  0-3 | 0.121 | 4.5±3.9  4.5  0-11 | 0.9±1.5  0.0  0-3 | **0.0323** |
| **Treatment** | | | | | | | | | | | | |
| Corticoids | 0 (0) | 0 (0) | 1.000 | 0 (0) | 0 (0) | 1.000 | 0 (0) | 0 (0) | 1.000 | 0 (0) | 0 (0) | 1.000 |
| Antibiotics | 3 (30) | 3 (43) | 0.6437 |  |  |  |  |  |  |  |  |  |
| Antipyretics | 8 (80) | 5 (71) | 1.000 |  |  |  |  |  |  |  |  |  |
| Antivirals | 1 (10) | 0 (0) | 1.000 |  |  |  |  |  |  |  |  |  |

BMI: body mass index; pSO2: oxygen saturation; SD: standard deviation.

*Chalder fatigue scale: 4 or more qualifying for "caseness."

**TABLE S3** Demographic and clinical characteristics of the trial population under placebo treatment

| Physiological variable | 1 days post-treatment | | 8 days post-treatment | | 90 days post-treatment | |
| --- | --- | --- | --- | --- | --- | --- |
|  | Men | Women | Men | Women | Men | Women |
| IP-10 | 0.002 | 0.01 | 0.013 |  |  |  |
| IL-8 |  | <0.001 |  |  |  |  |
| Eotaxin |  |  |  |  |  | 0.041 |
| SDF-1α | 0.047 |  |  |  |  |  |
| SCF | 0.001 | 0.046 |  |  |  |  |
| TRAIL | 0.002 | 0.003 |  |  |  |  |
| IL-IRα |  | 0.029 |  | 0.001 |  |  |
| Hemoglobin |  |  |  |  | 0.046 |  |
| ALT |  |  |  | 0.002 |  | 0.037 |
| Albumin |  | 0.0235 |  | 0.05 |  | 0.003 |
| D-dimer | 0.043 | <0.001 |  | <0.001 | 0.0224 | <0.001 |
| LDH |  |  |  |  |  | 0.043 |
| C reactive protein | 0.007 | 0.034 | 0.025 |  |  |  |
| ELR |  | 0.033 |  |  |  |  |
| pSO2 |  | 0.015 |  |  |  |  |
| Chronic Fatigue Syndrome |  |  |  |  |  | 0.045 |

**TABLE S4**. Statistical significance difference for each physiological value difference between groups (Placebo-PTIC) for men and women given by the p-value derived by the non-parametric Mann- Whitney- Wilcoxon rank.

|  | Placebo | | | | PTIC | | | |
| --- | --- | --- | --- | --- | --- | --- | --- | --- |
|  | Baseline | 1 | 8 | 90 | Baseline | 1 | 8 | 90 |
| Density | 0.13 | 0.12 | 0.08 | 0.10 | 0.20 | 0.22 | 0.08 | 0.07 |
| Transitivity | 0.48 | 0.50 | 0.41 | 0.48 | 0.55 | 0.67 | 0.42 | 0.39 |
| Small world index | 3.29 | 3.58 | 4.71 | 4.31 | 2.45 | 2.56 | 4.93 | 5.35 |

**TABLE S5.** Physiological network topology for placebo and PTIC groups at the baseline and 1, 8, and 90 post-treatment days.
