# Supplementary figures and images for: Polymerised type I collagen modifies the physiological network of post‐acute sequelae of COVID‐19 depending on sex: a randomised clinical trial
Source: Clin Transl Med. 2023 Oct 29;13(11):e1436. doi: 10.1002/ctm2.1436 (PMC10613754; doi:10.1002/ctm2.1436)

**Supplementary Material**


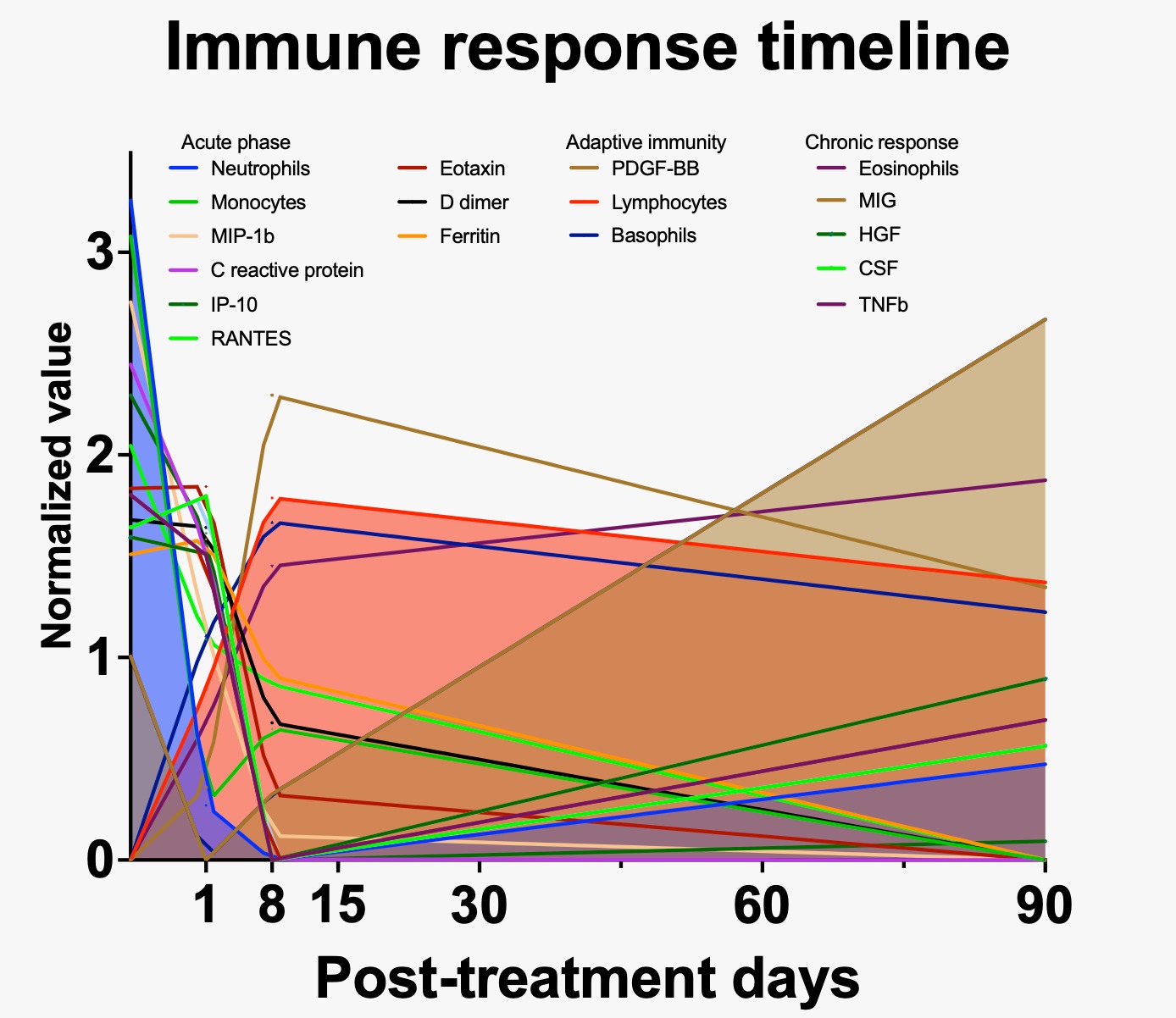


Figure S1: Inmune response timeline

Supplement: Supplementary file 2 — Supporting Information [file CTM2-13-e1436-s005.docx]
