# Supplementary material for: Polymerised type I collagen modifies the physiological network of post‐acute sequelae of COVID‐19 depending on sex: a randomised clinical trial
Source: Clin Transl Med. 2023 Oct 29;13(11):e1436. doi: 10.1002/ctm2.1436 (PMC10613754; doi:10.1002/ctm2.1436)
Supplement: Supplementary file 3 — Supporting Information [file CTM2-13-e1436-s001.docx]

**
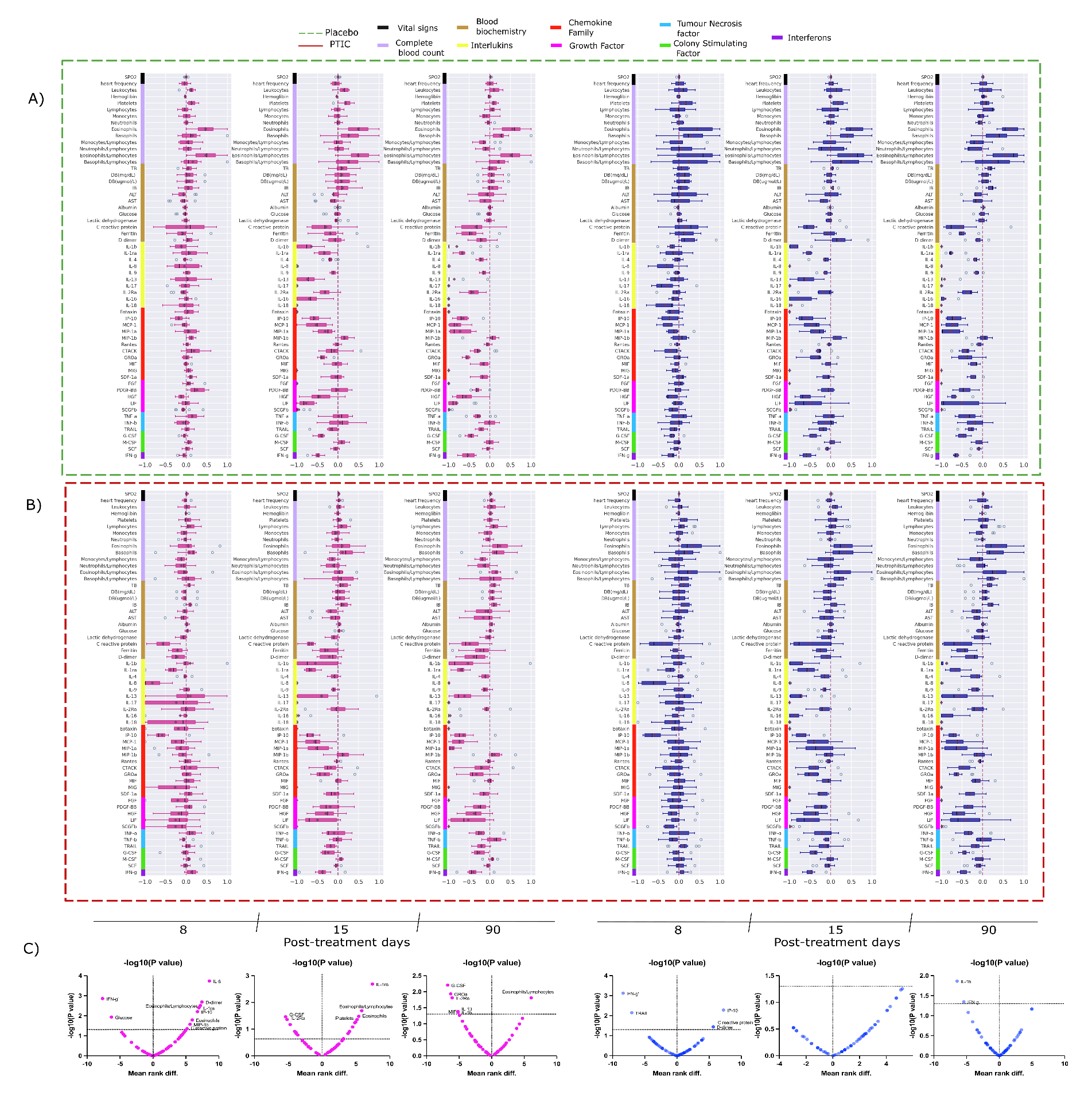
Supplementary Material**

Figure S2: Boxplot of the relative values of each physiological variable for A) the placebo group and B) the PTIC group, divided by sex, over successive days. C) Volcano plot of mean rank differences for specific physiological variables comparing placebo and PTIC groups for men (blue dots) and women (pink dots), respectively.
