# Supplementary material for: Polymerised type I collagen modifies the physiological network of post‐acute sequelae of COVID‐19 depending on sex: a randomised clinical trial
Source: Clin Transl Med. 2023 Oct 29;13(11):e1436. doi: 10.1002/ctm2.1436 (PMC10613754; doi:10.1002/ctm2.1436)
Supplement: Supplementary file 4 — Supporting Information [file CTM2-13-e1436-s004.docx]

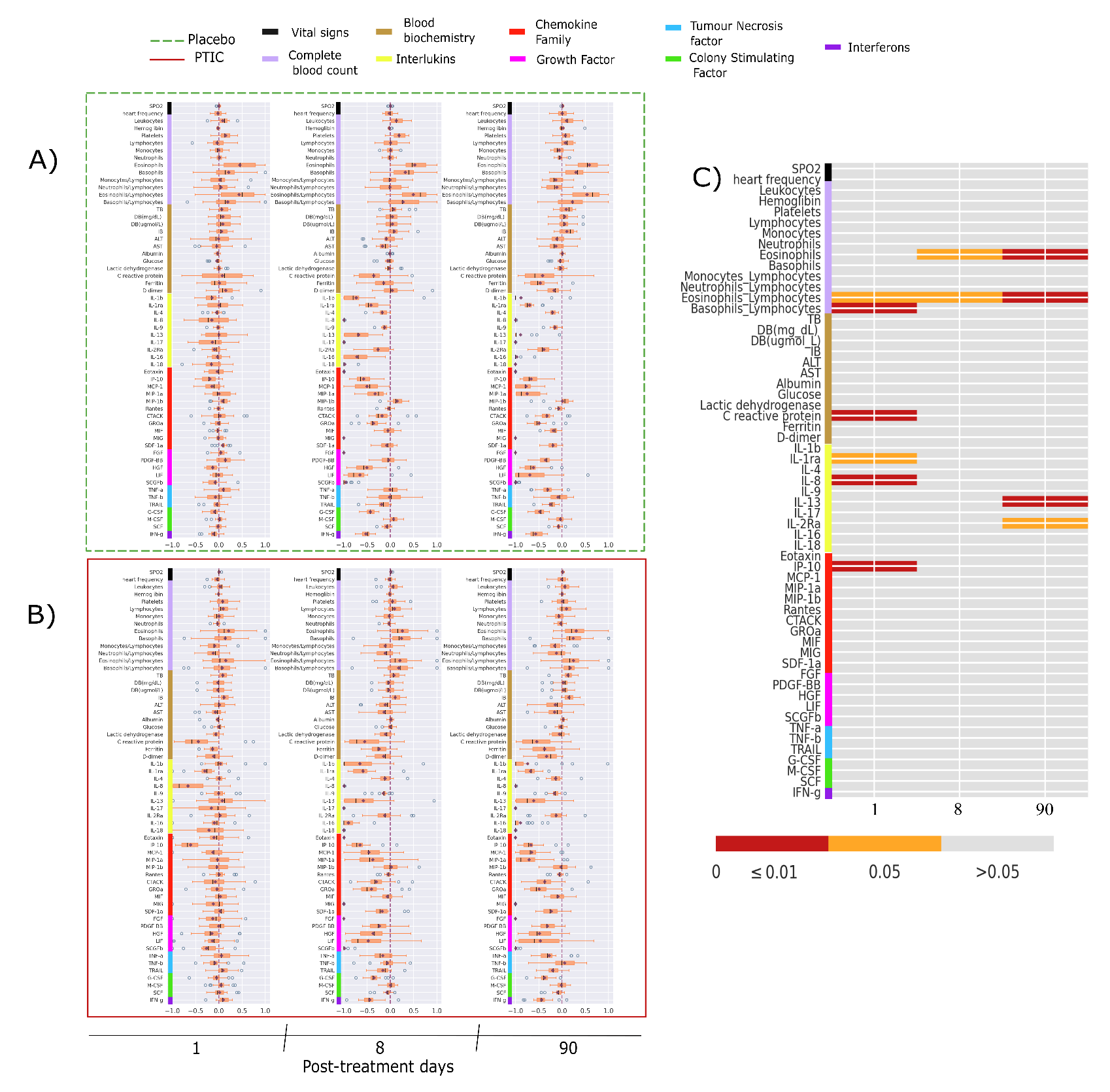
**Supplementary Material**

**Figure S3**: Boxplots of relative values for specific physiological variables at days 1, 8 and 90 post-treatment days, for A) the placebo group and B) the PTIC treatment group. Panel C) shows a color map of the p-values derived from a 2-way ANOVA with Bonferroni correction comparing the placebo and PTIC groups for specific physiological variables. Physiological variables are ordered according to functional groups (different colors in vertical line).
