# Supplementary material for: Polymerised type I collagen modifies the physiological network of post‐acute sequelae of COVID‐19 depending on sex: a randomised clinical trial
Source: Clin Transl Med. 2023 Oct 29;13(11):e1436. doi: 10.1002/ctm2.1436 (PMC10613754; doi:10.1002/ctm2.1436)
Supplement: Supplementary file 5 — Supporting Information [file CTM2-13-e1436-s003.docx]

**
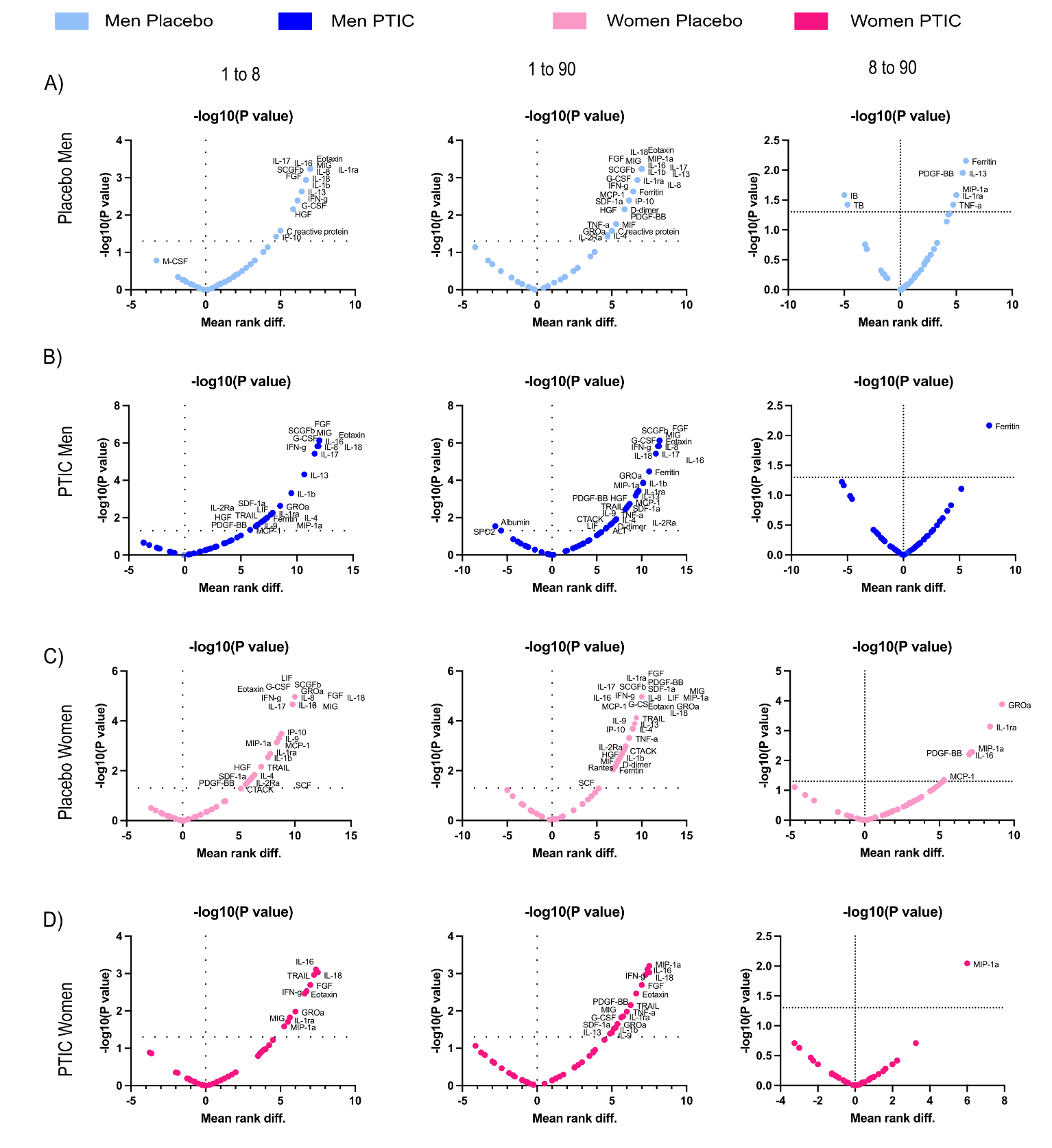
Supplementary Material**

**FIGURE S4** Volcano plots derived from relative values in the A)-C) placebo and B)-D) PTIC groups, differentiating between men (blue dots) and women (pink dots), respectively. The volcano plots show the p-value vs. mean ranks (difference between experimental days).
