# Supplementary material for: Polymerised type I collagen modifies the physiological network of post‐acute sequelae of COVID‐19 depending on sex: a randomised clinical trial
Source: Clin Transl Med. 2023 Oct 29;13(11):e1436. doi: 10.1002/ctm2.1436 (PMC10613754; doi:10.1002/ctm2.1436)
Supplement: Supplementary file 6 — Supporting Information [file CTM2-13-e1436-s006.docx]

**Supplementary Material**


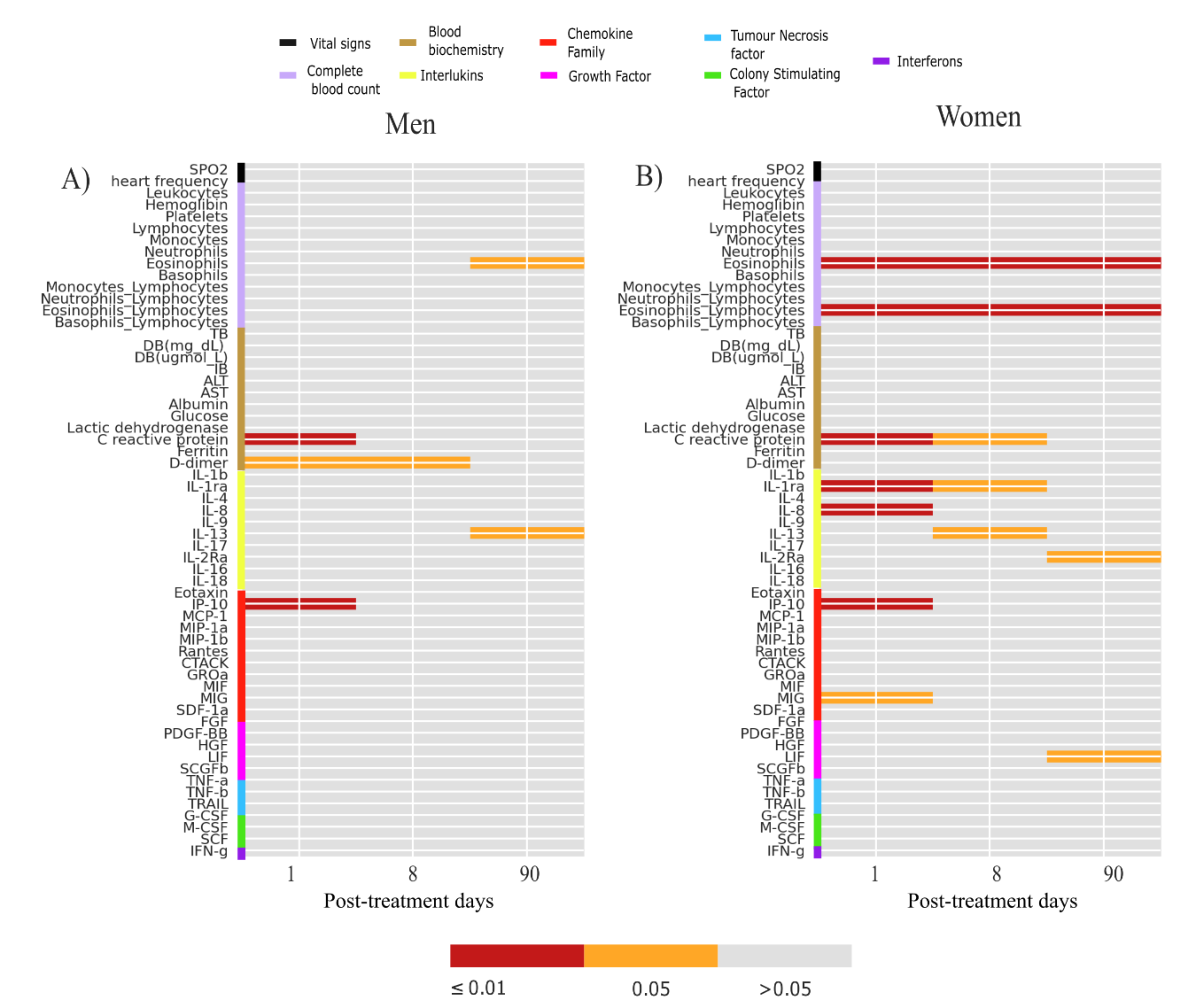


**FIGURE S5**: Colormap of p-values of statistical significance by according to 2-way ANOVA with Bonferroni correction of difference of physiological variables between placebo and PTIC groups. The plots distinguish between significance levels (p≤ 0.01 shown in red, p ≤ 0.05 shown in yellow, and non-significant shown in gray).
