# Supplementary material for: Polymerised type I collagen modifies the physiological network of post‐acute sequelae of COVID‐19 depending on sex: a randomised clinical trial
Source: Clin Transl Med. 2023 Oct 29;13(11):e1436. doi: 10.1002/ctm2.1436 (PMC10613754; doi:10.1002/ctm2.1436)

**Supplementary Material**

**FIGURE S6** Average physiological networks for A) the placebo group and B) the PTIC treatment group at baseline and for days 1, 8 and 90 post-treatment.
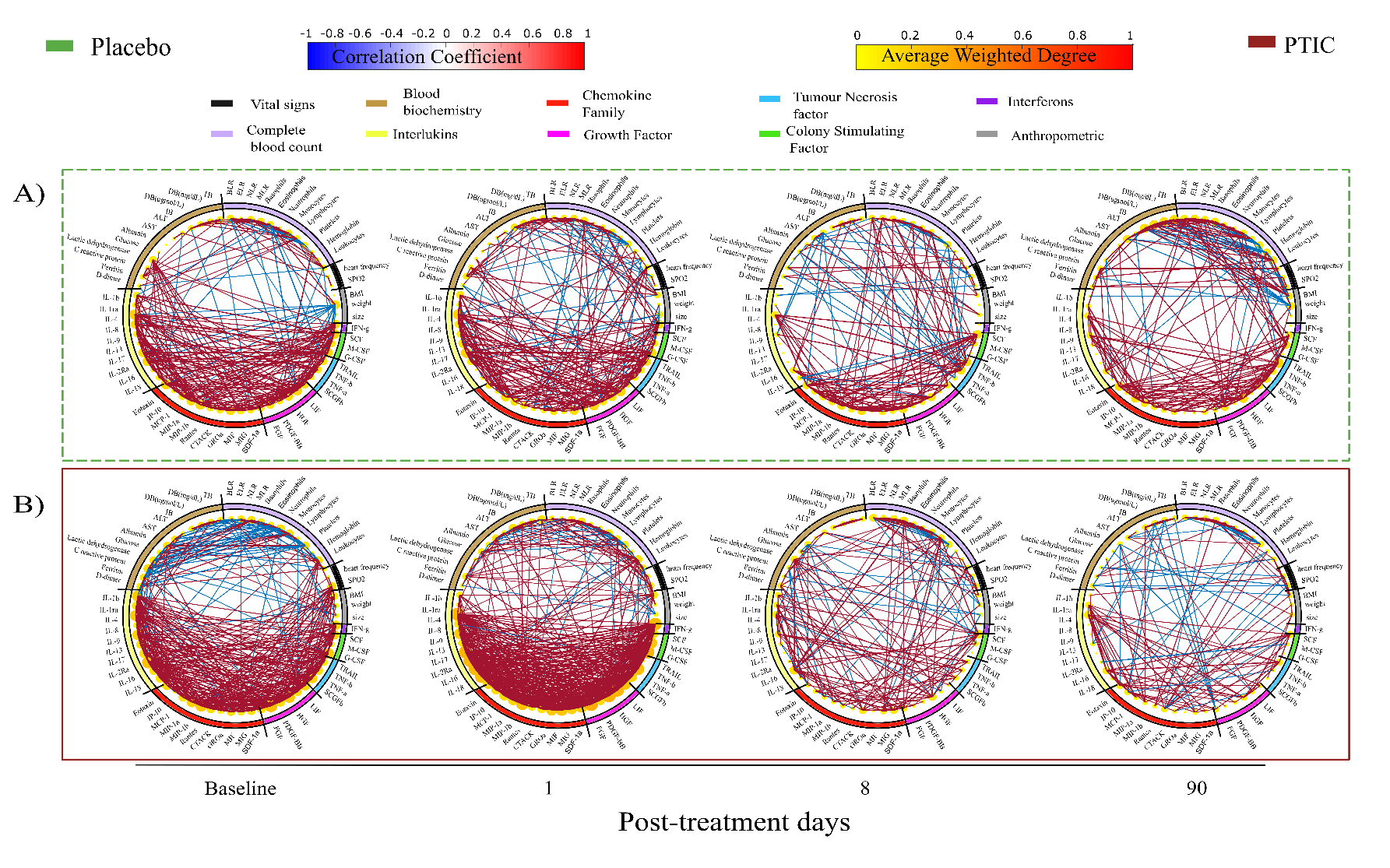

Supplement: Supplementary file 7 — Supporting Information [file CTM2-13-e1436-s002.docx]
